# Prevalence and Temporal Trends Analysis of Screening and Diagnostic Instruments in Posttraumatic Stress Disorder: Text Mining Study

**DOI:** 10.2196/33599

**Published:** 2021-11-17

**Authors:** Hui Zong, Binyang Hu, Yang Han, Zuofeng Li, Xiaoyan Zhang

**Affiliations:** 1 Research Center for Translational Medicine, Shanghai East Hospital School of Life Sciences and Technology Tongji University Shanghai China; 2 Philips Research China Shanghai China

**Keywords:** posttraumatic stress disorder, instruments, prevalence, clinical trials, text mining

## Abstract

**Background:**

Various instruments for patient screening and diagnosis have been developed for and applied in posttraumatic stress disorder (PTSD).

**Objective:**

This study comprehensively investigates the prevalence and temporal trends of the most widely used instruments in PTSD-related studies.

**Methods:**

A total of 1345 files of registered clinical trials from ClinicalTrials.gov and 9422 abstracts from the PubMed database from 2005 to 2020 were downloaded for this study. The instruments applied in clinical trials were manually annotated, and instruments in abstracts were recognized using exact string matching. The prevalence score of an instrument in a certain period was calculated as the number of studies divided by the number of instances of the instrument. By calculating the yearly prevalence index of each instrument, we conducted a trends analysis and compared the trends in index change between instruments.

**Results:**

A total of 4178 instrument synonyms were annotated, which were mapped to 1423 unique instruments. In the 16 years from 2005 to 2020, only 10 instruments were used more than once per year; the 4 most used instruments were the PTSD Checklist, the Clinician-Administered PTSD Disorder Scale, the Patient Health Questionnaire, and the Beck Depression Inventory. There were 18 instruments whose yearly prevalence index score exceeded 0.1 at least once during the 16 years. The changes in trends and time points of partial instruments in clinical trials and PubMed abstracts were highly consistent. The average time duration of a PTSD-related trial was 1495.5 days or approximately 4 years from submission to ClinicalTrial.gov to publication in a journal.

**Conclusions:**

The application of widely accepted and appropriate instruments can help improve the reliability of research results in PTSD-related clinical studies. With extensive text data obtained from real clinical trials and published articles, we investigated and compared the usage of instruments in the PTSD research community.

## Introduction

Posttraumatic stress disorder (PTSD) is a mental health condition triggered by experiencing or witnessing a traumatic event [1,2]. Over 70% of adults worldwide have experienced a traumatic event at least once in their lifetime, with 30.5% have experiencing 4 or more events [3]. The most commonly reported traumatic events for individuals are the unexpected death of a loved one, witnessing death or serious injury, being robbed, and life-threatening automobile accidents [4]. Rapid and accurate assessment facilitates timely diagnosis and early intervention in PTSD. Assessment tools comprise screening and diagnostic instruments, which vary in their format (self-reporting or structured interviews) depending on the population, target symptoms, or actions for which they are designed. With the advancement of modern medicine, many instruments have been developed and applied in scientific research and clinical trials. However, choosing the appropriate instrument for a PTSD study can be challenging without comprehensive comparison or evaluation.

Several studies have investigated and compared commonly used instruments in PTSD. In a previous study [5], researchers conducted a web-based survey on 277 traumatic stress professionals to assess traumatic event exposure and posttraumatic effects and revealed 7 commonly used instruments, including the Post-traumatic Stress Diagnostic Scale, the Trauma Symptom Inventory, the Life Events Checklist, the Clinician-Administered PTSD Scale (CAPS), the PTSD Checklist (PCL), the Impact of Event Scale-Revised, and the Trauma Symptom Checklist. In another study, researchers described the reliability and validity of common self-report instruments and structured clinical interviews used to assess depression [6] and PTSD after sepsis [7]. Some researchers also compared different versions of the PCL spanning the transition between the Diagnostic and Statistical Manual of Mental Disorders, Fourth Edition (DSM-IV) and DSM-V [8]. The above studies demonstrated the importance of determining the most widely used instruments. However, the number of participants, the institutions they belong to, the number of instruments included, and subjective or memory factors may have introduced bias in the results.

With the exponential growth of biomedical literature, text mining becomes increasingly promising for biomedical research, especially in the fields of public health and biomedical informatics. Extracting potentially useful information using keyword matching or advanced methods and investigating the prevalence trends of specific topics can help to gain better insight into a particular field and discover inconspicuous changes. Analysis of prevalence trends is a widespread practice of collecting information and attempting to spot trends in the information, such as cultural trends [9], cognitive distortion prevalence [10], research topic trends [11], and top popular questionnaires [12]. These studies have shed light on large-scale text data analyses for examining prevalence and trends.

There are growing numbers of published articles and ongoing registered clinical trials involving PTSD, in which several instruments have been applied to assess the symptoms, emotions, feelings, and actions of the participants [6]. Investigating and comparing the prevalence and temporal trends of these instruments can help determine the conventional assessment tools used in this field. In addition, this valuable knowledge can be provided to researchers when developing assessment criteria in clinical studies, which can particularly benefit clinicians and researchers who are new to the field.

In this study, we conducted a comprehensive investigation on the prevalence trends of the most widely employed instruments using extensive text data from real clinical trials and published articles related to PTSD. It may help reveal the conventional assessment tools used for evaluating PTSD and provide valuable knowledge that might not be otherwise apparent. We believe the prevalence of instruments is an important index in measurement selection, and knowledge in this regard can serve as a reference for designing studies and trials.

## Methods

### Data and Annotation

**Figure 1:**
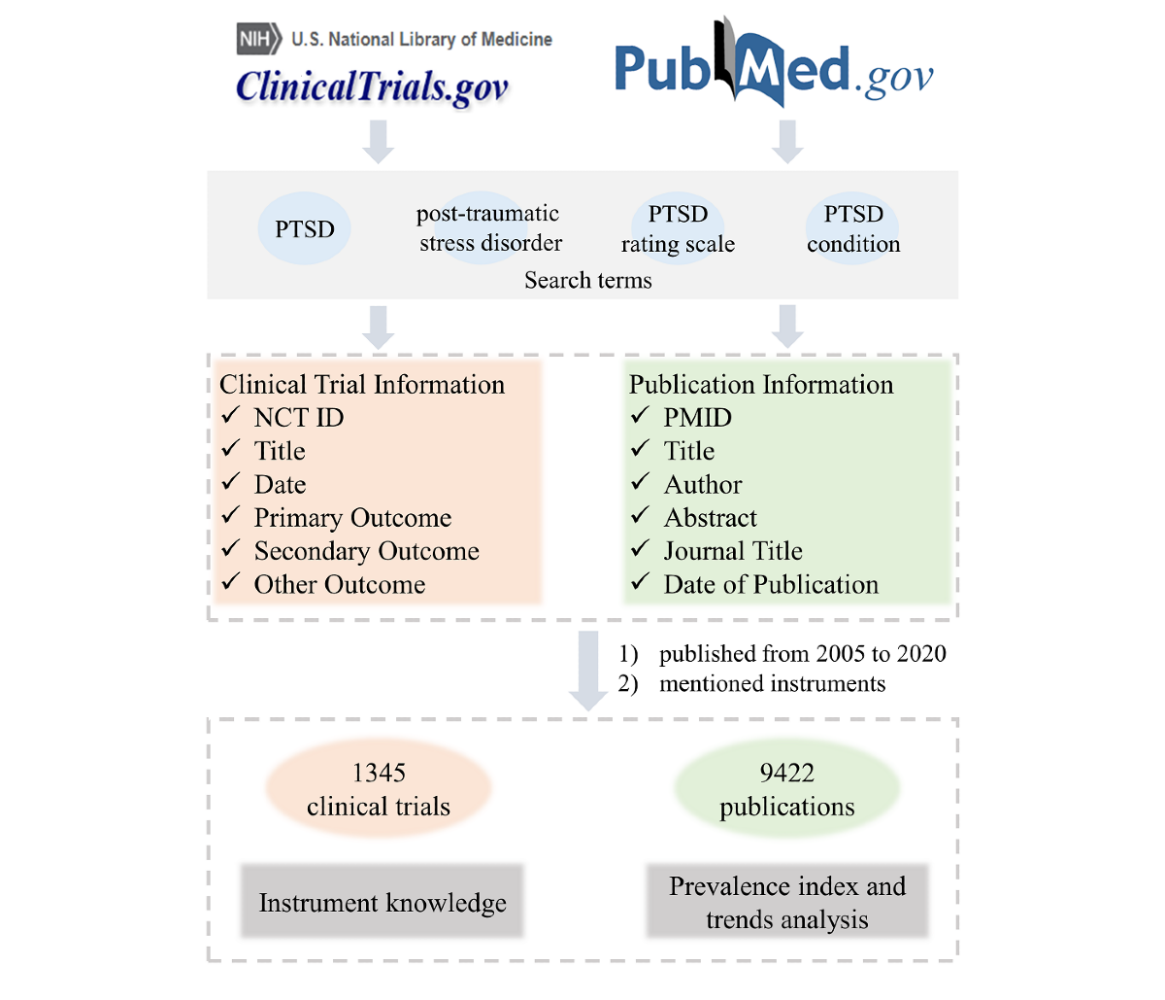
Flow diagram showing collection process of clinical trials and PubMed abstracts included in this study. NCT ID: National Clinical Trials identifier; PMID: PubMed identifier; PTSD: posttraumatic stress disorder.

A total of 2502 registered clinical trials were accessed and downloaded from ClinicalTrials.gov by searching with keywords “PTSD,” “PTSD rating scale,” “PTSD condition,” and “post-traumatic stress disorder,” as shown in Figure 1. Important details, such as the clinical trial identifier, title, brief summary, date, study description, and outcome measures, were extracted and manually reviewed to determine whether the clinical trials were related to PTSD. A custom Python script (Python Software Foundation) was used to retrieve clinical trials, download registration files, and extract previous information. We established 2 exclusion criteria during this step. First, trials that mentioned PTSD but focused on other diseases were eliminated; for example, a trial on psychogenic nonepileptic seizures that employed the Davidson Trauma Scale (DTS) and mentioned PTSD when introducing the DTS was excluded. Second, trials that were related to PTSD but did not include any instruments in their outcome measures were eliminated; for instance, a trial that focused on the treatment of PTSD with guanfacine was excluded since specific assessment instruments were not mentioned.

Following the confirmation of relevance, the instruments in each study were annotated from the designed outcome measures. Instruments were restricted to rating scales, self-report inventories, and structured interviews, such as the CAPS [13] and the PCL [14]. Laboratory tests (such as heart rate variability), mentions of symptoms without a clear statement on the instrument applied (eg, weekly number of nightmares and depression symptoms), and other measures were ignored during the annotation. The annotations were performed using brat [15], a widely used web-based tool for text annotation in text mining.

Based on our annotations and the 2 exclusion criteria, 1120 clinical trials were excluded from our data set. As there were only 37 trials found from 1999 to 2004 and the small number of trials made it difficult to calculate the prevalence index, we excluded trials prior to 2005 in this study. In total, 1345 trials were included in our final clinical trial data set for prevalence analysis, and their first submission dates on ClinicalTrials.gov ranged from March 2005 to December 2020.

### Instrument Knowledge Construction

A unified name mapping system was built to map the different original instrument names in the text to their corresponding normalized full names to distinguish different instruments and their abbreviations to indicate different versions of each instrument. For example, the PTSD Checklist–Civilian Version and the PTSD Checklist–Military Version were mapped to the normalized full name PTSD Checklist, whereas the abbreviations were assigned as PCL-C and PCL-M, respectively.

### Prevalence Index

The prevalence of an instrument is indicated by its usage frequency, which is calculated as the total number of studies in a given period divided by the number of times an instrument is used in that period.

The strength of this index is its capacity to reduce the noise caused by different numbers of studies during different time periods. It quantifies the prevalence and enables comparison of the prevalence across different time periods. In this study, the prevalence index for each instrument was calculated for each year. For example, there were 34 clinical studies in 2006 and the CAPS was used 20 times during that year. Therefore, the prevalence index of the CAPS in 2006 was 0.5882 (20/34). In 2008, the CAPS was employed in 34 of 57 studies and the prevalence index was 0.6071 (34/56). Although the total number of studies and the number of times each instrument was applied varied, there was no significant change in the prevalence of the CAPS.

To conduct trends analysis on instruments, we calculated and visualized the yearly prevalence index for each instrument and then compared the index change trends between instruments. After trends analysis, we were able to determine whether an instrument was still popular or its usage was decreasing, which instruments were more commonly used, and those that will be widely used in future studies.

### Validation With PubMed

Considering the bias and inadequacy introduced by only using registered clinical trials in ClinicalTrials.gov, we retrieved and downloaded PTSD-related abstracts from PubMed using the previously mentioned keywords. A custom Python script, using the Entrez application program interface, helped us retrieve the PubMed IDs and extract publication information. A similar trends analysis was conducted on instruments mentioned in those publications to validate the analysis results from clinical trials. The instruments mentioned in the abstracts were automatically recognized by exact string matching using the various instrument names obtained during manual annotation. Abstracts that did not mention any instrument were excluded. In total, 9422 abstracts were included for prevalence and temporal trends analysis, as shown in Figure 1.

To evaluate the risk of a clinical study’s measures being obsolete, the time durations between the submission and publication dates of a clinical trial were also compared. For a published study, the time duration was defined as the interval between the first posted date in ClinicalTrials.gov and the publication date in PubMed. If there were multiple papers published based on one clinical trial, the earliest publication date was used. This comparison was performed to infer the potential influence of knowledge updates on clinical studies.

## Results

### Overall Trends

A total of 4178 instrument synonyms were annotated, which were mapped to 1423 unique instruments. The number of trials, the number of applied instruments, and the number of applied unique instruments in each year, as well as the average number of instruments applied in one trial, are provided in Table 1. It should be noted that the number of scales applied each year shows an increasing trend over time.

From 2005 to 2020, only 10 instruments were used more than once per year (Table 2), 17 instruments were used ≥50 times, and 1255 instruments were employed less than 10 times in total. The most commonly used instruments were the PCL, the CAPS, the Patient Health Questionnaire (PHQ), and the Beck Depression Inventory (BDI). The list of instruments and their prevalence trends are available online [16].

**Table 1:** Statistical data for each year.

Year	Trials, n	Applied instruments, n	Unique instruments applied, n	Instruments used in one trial, mean
2005	44	170	63	3.86
2006	34	186	97	5.47
2007	34	112	57	3.29
2008	56	207	92	3.70
2009	58	228	107	3.93
2010	60	217	115	3.62
2011	68	272	133	4.00
2012	104	422	187	4.06
2013	87	408	209	4.69
2014	87	389	190	4.47
2015	89	424	223	4.76
2016	99	398	201	4.02
2017	106	538	277	5.08
2018	112	586	276	5.23
2019	136	725	318	5.33
2020	171	842	326	4.92

**Table 2:** Instruments applied more than once per year and their associated total prevalence index (2005-2020).

Instrument	Times applied, n	Total prevalence index^a^
PTSD^b^ Checklist	541	0.4022
Clinician-Administered PTSD Scale	495	0.3680
Patient Health Questionnaire	202	0.1502
Beck Depression Inventory	175	0.1301
Pittsburgh Sleep Quality Index	110	0.0818
Short Form Health Survey	108	0.0803
Impact of Event Scale	91	0.0677
Clinical Global Impression	84	0.0625
Hospital Anxiety and Depression Scale	71	0.0528
Sheehan Disability Scale	66	0.0491

^a^The total prevalence index was calculated by dividing the number of times an instrument was used from 2005 to 2020 by the total number of clinical trials in those 16 years.

^b^PTSD: posttraumatic stress disorder.

**Figure 2:**
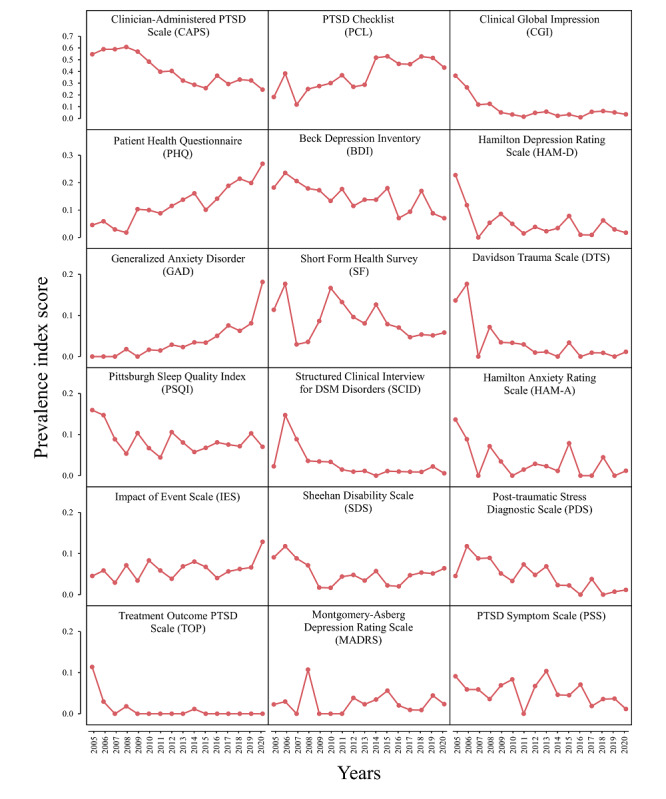
Prevalence indexes for the top 18 instruments whose indexes exceeded 0.1 at least once between 2005 and 2020. DSM: Diagnostic and Statistical Manual of Mental Disorders; PTSD: posttraumatic stress disorder.

To analyze the temporal trends of the instruments, the prevalence index for each instrument was calculated for each year. There were 18 instruments whose prevalence index exceeded 0.1 at least once in the 16 years, as observed in Figure 2. Among the 18 instruments with distinct variation trends, the prevalence index of the CAPS took 16 years (2005 to 2020) to decrease from 0.5455 to 0.2456, which is a relatively long period. In the 11 years from 2005 to 2015, the usage rate of the PCL increased from 0.1819 to 0.5281. The prevalence index of the PHQ increased 4 times after 2008, and the use of the Clinical Global Impression (CGI) decreased by approximately 68% (from 0.3636 to 0.1176) in 2 years beginning in 2005. The prevalence indexes of the Structured Clinical Interview for DSM Disorders (SCID), DTS, and PHQ also decreased by more than 50% over 2 or 3 years beginning in 2005 or 2006. The results showed that even the usage rates of the top instruments in this field were not very high. Scales such as the CAPS, the CGI, the BDI, and the Hamilton Depression Rating Scale (HAM-D) were popular in the early years, but their usage rate decreased over time.

### Trend Analysis Based on the Assessment Targets

It is difficult to determine the latent information behind temporal trends when all available instruments are considered. Therefore, we compared and analyzed the changing trends among the top instruments according to their assessment targets. By comparing the usage trends of rating scales with similar functions, changes in the conventional usage of assessment tools can be revealed.

**Figure 3:**
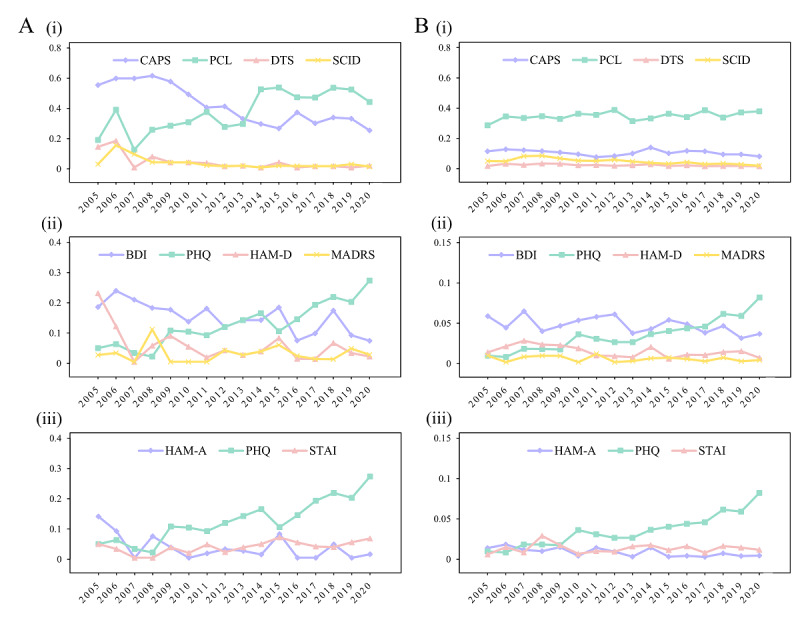
Temporal trends comparison of the instruments based on the same assessment target: (A) based on the clinical trials data and (B) based on the published abstracts data set. (i) Indicates the comprehensive scales; (ii) indicates the depression symptoms scales; (iii) indicate the anxiety symptoms scales. In both data sets, the PTSD Checklist (PCL) and the Patient Health Questionnaire (PHQ) show steady upward trends, whereas the use of the Clinician-Administered PTSD Disorder Scale (CAPS) and Beck Depression Inventory (BDI) is decreasing. DTS: Davidson Trauma Scale; HAM-A: Hamilton Anxiety Rating Scale; HAM-D: Hamilton Depression Rating Scale; MADRS: Montgomery-Asberg Depression Rating Scale; SCID: Structured Clinical Interview for Diagnostic and Statistical Manual Disorders; STAI: State-Trait Anxiety Inventory.

The CAPS, PCL, DTS, and SCID were categorized as “comprehensive rating scales” that assess the symptoms of PTSD based on the DSM. The BDI, PHQ, HAM-D, and Montgomery-Asberg Depression Rating Scale were classified into the “depression” group. The Hamilton Anxiety Rating Scale (HAM-A), PHQ, and State-Trait Anxiety Inventory (STAI) were classified under the “anxiety” scale group. The usage rates of each group are shown in Figure 3A.

There were 9422 PTSD-related papers published from 2005 to 2020 with instruments mentioned in their abstracts. The top instruments were similar to those identified in the clinical trial data set, with some changes in their rank. In the PubMed data set, the PCL was the most used instrument (appearing 3244 times) and the Impact of Event Scale was the second most common instrument (appearing 1057 times). The trends in the prevalence indexes of the 3 groups are presented in Figure 3B, demonstrating that the changes in trends and time points are highly consistent with the results of the clinical trial data set.

### Trial Time Duration and Changes in the Prevalence Rate

**Figure 4:**
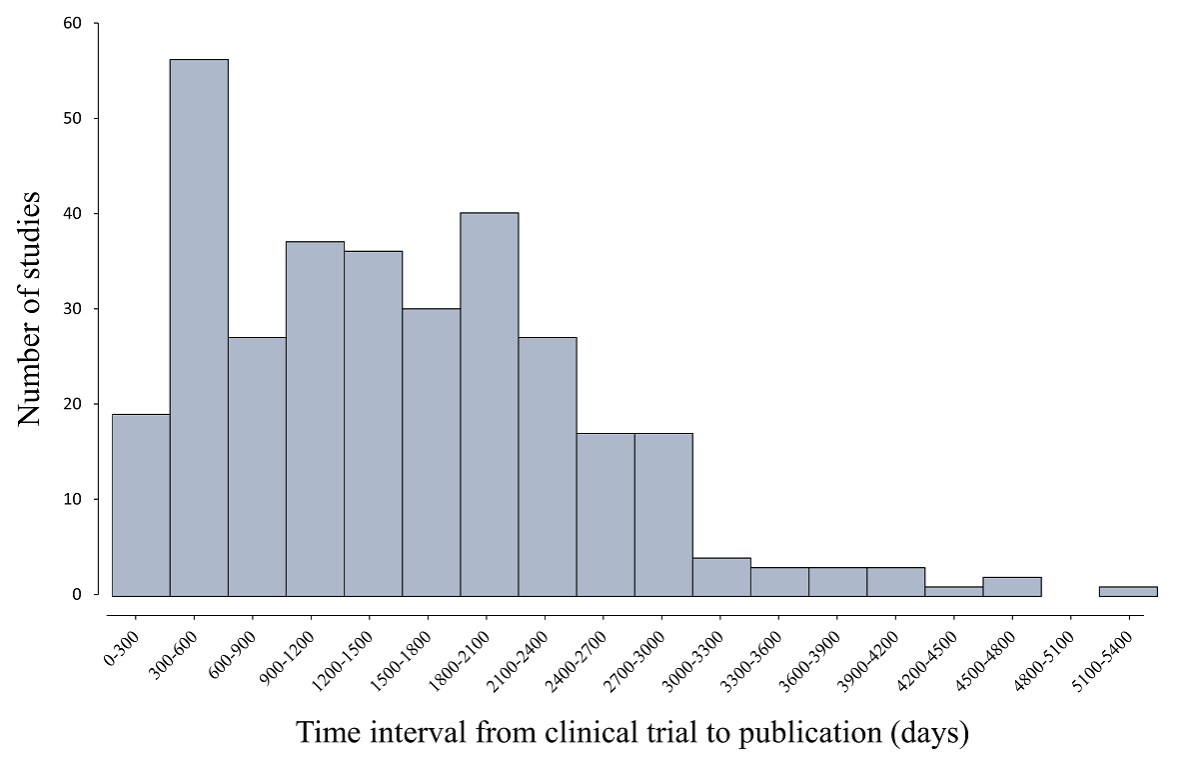
Distribution of the publication time durations of the 323 clinical studies ranging from 78 to 5339 days.

The average time duration of the clinical trials was compared with the time required for the prevalence of an instrument to significantly increase or decrease. We retrieved 487 papers from the PubMed database, which were derived from 323 clinical trials. The interval between the first submission date in ClinicalTrials.gov and the publication date of the earliest paper was regarded as the time duration. The results in Figure 4 show that the average time duration is 1495.5 days or approximately 4 years. Most of the trials (n=253) required 300 to 2400 days to publish a paper after they started, and the average time duration of these 253 trials was 1275 days (median 1281 days). In the 323 trials, the CAPS and the PCL were the most popular instruments, and both were employed 116 times.

## Discussion

### Principal Findings

Our results demonstrate that there are many available instruments with generally low usage rates, indicating that there is no conventional assessment tool for PTSD. It is meaningful to reveal the trends and knowledge based on which researchers select assessment tools to use in clinical trials.

As we observed the decreased prevalence of some top instruments, two reasons seemed appropriate to explain this phenomenon. One reason is the reduction in the number of instruments in each clinical trial. For example, 1 clinical trial may have used approximately 10 instruments early in the study, 7 of which were the top instruments; however, currently, the same clinical trial only uses 5 instruments, 3 of which are top instruments. This reduction can lead to decreased prevalence owing to the method used to calculate the prevalence index. The other probable reason is that a certain number of new instruments were introduced into the field each year, diluting the prevalence of the top instruments. To verify these possibilities, the number of instruments applied in each year and the average number of instruments applied in 1 trial were calculated. The latter reason was supported by the results presented in Table 1. It seems that the number of new, but not widely used, scales introduced into the field each year diluted the prevalence of all the instruments and reduced the usage of a particular conventional instrument.

Analysis of the temporal trends showed that the CAPS and the PCL were the most widely applied, comprehensive instruments, and their high usage rate demonstrated that most of the clinical trials required comprehensive diagnostic instruments. The popularity of the CAPS decreased (from 0.5455 in 2005 to 0.2456 in 2020), whereas that of the PCL increased (from 0.1818 in 2005 to 0.4327 in 2020) over time, which was also validated using the PubMed data. To determine the reason for this phenomenon, we retrieved and reviewed studies related to the CAPS and the PCL. As Fonkoue et al [17] point out, the CAPS and the PCL have excellent psychometric properties with high interrater reliability, test-retest reliability, and internal consistency [18]. The scores of the CAPS and the PCL were also highly correlated and showed high diagnostic agreement [19,20]. The most likely cause of this phenomenon could be the different formats of these instruments, rather than their quality. The CAPS employs a structured interview that can only be administered by trained and experienced clinicians, whereas the PCL is a more convenient self-report assessment instrument that requires less time and resources.

As for the depression assessments, the results from both data sets indicate that although the BDI is still the most widely used assessment instrument (increasing from approximately 0.07 to 0.24 over the 16-year period), usage of the PHQ is increasing (from 0.05 to 0.27) and it may become the most prevalent instrument to assess depression symptoms. Compared with the STAI and the HAM-A, the PHQ was also the most commonly used instrument for anxiety symptoms. These results show that since 2009, the PHQ has become a widely accepted instrument to assess depression and anxiety. One possible reason for these findings may be that the PHQ-9 is a relatively new, simple, and freely available instrument [21].

There were also rating scales that had no alternatives among the most applied instruments, such as the Pittsburgh Sleep Quality Index to assess sleep, the Short Form Heath Survey to assess an individual’s health status, and the Impact of Event Scale to assess the influence of traumatic events. These instruments are regarded as the accepted scales for their respective assessment targets.

Compared with the time duration required for clinical research to be published (4 years on average), variations in the prevalence of instruments occur rapidly. The popularity of instruments such as the PHQ, CGI, SCID, DTS, and HAM-A changed by more than 50% in less than 3 years. In contrast, trends in the prevalence of popular instruments such as the CAPS and the PCL indicate that they are more stable. Considering the reliability of research outcomes as well as the stability and high acceptance of study measures, employing the scales that are more prevalent or trending upward is recommended when there are several alternatives that can meet research interests equally. This can also help to build a consensus regarding the assessment tools used in the field of PTSD.

Although we have tried to include as many PTSD-related clinical trials as possible in this study, there are some limitations to using ClinicalTrials.gov as a data resource: (1) this data source may not be able to represent all researchers studying PTSD; and (2) the studies included in ClinicalTrials.gov change over time (such as the inclusion of more small-scale trials), which may also result in temporal changes.

To overcome these limitations and validate the results obtained from ClinicalTrials.gov, we used the large-scale PubMed data set to conduct similar trend analyses. We found that the results obtained using the PubMed data set were highly consistent with those obtained from the ClinicalTrials.gov data set. In this manner, the results and conclusions of our approach can be reciprocally verified by the clinical trials and their corresponding publications. Although we aimed to conduct a trends analysis on all presently used instruments, this task was difficult to complete. Most of the instruments were applied for less than 6 years with a low usage rate, which resulted in many zero values and fluctuating trends in the prevalence indexes. To achieve a reliable conclusion, the temporal trends analysis only focused on the most popular instruments. Therefore, some instruments known to be relevant to PTSD are not mentioned in our results and discussions, such as the Composite International Diagnostic Interview (CIDI), a diagnostic measure for PTSD that was only used 5 times from 2005 to 2020. There are 12 years during which all the clinical trials in that year did not employ the CIDI in their studies (ie, prevalence index=0). The prevalence indexes for the CIDI in 2007, 2011, 2014, and 2015 were 0.0589, 0.0147, 0.0115, and 0.0112, respectively. Other relevant instruments such as the Posttraumatic Stress Syndrome Inventory and Short Inventory of Problems are not mentioned in this paper owing to the abovementioned reason.

In the future, more accurate natural language processing methods should be developed to recognize and extract instruments automatically from more databases. More integrated and comprehensive knowledge should also be collected. Based on this data and knowledge, comprehensive studies on instruments focusing on one condition can be conducted. The study objective, target population, and functions of the instruments should also be considered when recommending an instrument. A system that can automatically recommend suitable instruments for a certain study using statistical methods and indexes can be developed.

### Conclusions

Using widely accepted and applied instruments can help improve the reliability of research results in PTSD-related clinical studies. Considering the long duration of each study as well as the large variety of study instruments, it is challenging for researchers or clinicians to select the most appropriate instrument according to updated knowledge or trends. In this study, we investigated the prevalence indexes of various PTSD-related instruments and conducted a temporal trend analysis for PTSD-related studies using data from clinical trials and PubMed. Our work aimed to determine the most prevalently used instruments in PTSD-related clinical studies while considering the assessment target and to reveal knowledge and trends. Furthermore, we discuss the reasons for these trends to provide updated information and supportive knowledge to researchers to help reach a consensus regarding the use of assessment tools. Our results also demonstrate the feasibility of conducting a temporal trends analysis on clinical studies and its potential to support research design and implementation.

## Abbreviations

BDI: Beck Depression Inventory
CAPS: Clinician-Administered PTSD Scale
CGI: Clinical Global Impression
CIDI: Composite International Diagnostic Interview
DSM: Diagnostic and Statistical Manual of Mental Disorders
DTS: Davidson Trauma Scale
HAM-A: Hamilton Anxiety Rating Scale
HAM-D: Hamilton Depression Rating Scale
PCL: PTSD Checklist
PHQ: Patient Health Questionnaire
PTSD: posttraumatic stress disorder
SCID: Structured Clinical Interview for DSM Disorders
STAI: State-Trait Anxiety Inventory
